# Small-Area Estimation of the Probability of Toxocariasis in New York City Based on Sociodemographic Neighborhood Composition

**DOI:** 10.1371/journal.pone.0099303

**Published:** 2014-06-11

**Authors:** Michael G. Walsh, M. A. Haseeb

**Affiliations:** 1 Department of Epidemiology and Biostatistics, School of Public Health, State University of New York, Downstate Medical Center, Brooklyn, New York, United States of America; 2 Departments of Cell Biology, Pathology and Medicine, College of Medicine, State University of New York, Downstate Medical Center, Brooklyn, New York, United States of America; The George Washington University Medical Center, United States of America

## Abstract

Toxocariasis is increasingly recognized as an important neglected infection of poverty (NIP) in developed countries, and may constitute the most important NIP in the United States (US) given its association with chronic sequelae such as asthma and poor cognitive development. Its potential public health burden notwithstanding, toxocariasis surveillance is minimal throughout the US and so the true burden of disease remains uncertain in many areas. The Third National Health and Nutrition Examination Survey conducted a representative serologic survey of toxocariasis to estimate the prevalence of infection in diverse US subpopulations across different regions of the country. Using the NHANES III surveillance data, the current study applied the predicted probabilities of toxocariasis to the sociodemographic composition of New York census tracts to estimate the local probability of infection across the city. The predicted probability of toxocariasis ranged from 6% among US-born Latino women with a university education to 57% among immigrant men with less than a high school education. The predicted probability of toxocariasis exhibited marked spatial variation across the city, with particularly high infection probabilities in large sections of Queens, and smaller, more concentrated areas of Brooklyn and northern Manhattan. This investigation is the first attempt at small-area estimation of the probability surface of toxocariasis in a major US city. While this study does not define toxocariasis risk directly, it does provide a much needed tool to aid the development of toxocariasis surveillance in New York City.

## Introduction

Neglected infections of poverty (NIP) are increasingly recognized as important contributors to morbidity in marginalized populations in the United States (US) [1]. Unfortunately, much of the public health community has not considered such infections as relevant to the growing problem of health disparity in the US. As a result, these infections remain neglected. Toxocariasis may constitute the most important NIP experienced in the US due to its relatively high estimated prevalence nationwide, and, more importantly, because of chronic health problems that have been identified as long-term sequelae of this infection [2]. Previous reports from the National Health and Nutrition Examination Survey (NHANES) have estimated the seroprevalence of toxocariasis in the United States to be 14% overall, with substantial differences across ethnicity (21.2% among African-Americans, 10.7% among Mexican-Americans, and 12.0% among Whites) [3]. This singular source of extensive country-wide surveillance data identified a prevalence of toxocariasis that is concerning given the potential for chronic morbidity. For example, aside from the relatively rare ocular and visceral complications attending acute infection, diminished lung function [4] and poor cognitive development [5]–[7] have also been identified to far greater extent with respect to chronic infection. In addition, there remain substantive gaps in our knowledge of the local spatial distribution of toxocariasis in the US due to the lack of local surveillance. A previous study modelled the distribution of *Toxocara* seroprevalence at the county level across the whole of the US. The investigators found that toxocariasis was concentrated in the Northeast and South regions, with significant spatial variability throughout [8]. The study was well conceived and provided useful new information within the scope of its aims. Nevertheless, at the county level, the scale was still relatively small and could not provide insight into the risk surface at the larger, more local, scales of cities or neighborhoods within cities. Moreover, a large scale probability surface can incorporate the unique local demographic characteristics that may more accurately describe infection occurrence in small-area settings. Given the extreme paucity of local surveillance data for toxocariasis in urban settings, which vary significantly with respect to population composition, the current investigation sought to estimate the predicted probability surface for toxocariasis in the urban landscape of New York City. Data from a national population-based survey were used to model and estimate the probability of toxocariasis in each census tract in New York City based on the distinct sociodemographic composition of each tract.

## Materials and Methods

Using data from the Third National Health and Nutrition Examination Survey (NHANES III), this study first modelled toxocariasis and associated risk factors. This large population-based survey was conducted between the years of 1988 and 1994 by the National Center for Health Statistics at the Centers for Disease Control and Prevention, US. Methods describing this national survey have been previously published [9], [10], but will be summarized briefly below. The survey was designed to obtain nationally representative information on the health and nutrition status of the general US population through interviews and physical examinations. Eighty-six percent of selected individuals participated in the questionnaire interview, and 78% participated in the examination component, which included the collection of blood samples subsequently stored and used for detection of *Toxocara* antibodies. Sociodemographic data were collected in either the Mobile Examination Center or at the participants’ homes. As described in the NHANES laboratory documentation [10], [11], *Toxocara* antibodies were measured with an enzyme immunoassay (EIA) developed at the Centers for Disease Control and Prevention. This assay used an excretory/secretory antigen of *T. canis*. However, the assay was not able to distinguish between *T. canis* and *Toxocara cati* antibodies [3], and so this report simply refers to *Toxocara* spp. infection throughout the text. Level of education was used as a robust indicator of socioeconomic status and was categorized into three groups. The education level of individuals who did not complete high school was classified as “low”, the level of those who completed high school, but not university, was considered “middle”, and the level of those who completed university was considered “high”. Income level was collected on all participants in the survey, however we did not use this as an indicator of socioeconomic status because categories defining low, middle, and high income from 16 years prior could not be appropriately applied to income categories defining low, middle, and high income in census tracts in 2010. Ethnicity was determined by self-report and was comprised of four categories: African-American, Latino, White, and Other. The Latino category was comprised of both Mexican-Americans and other Latino ethnicities from Central and South America and the Caribbean. Immigrant status reflects the participant’s place of nativity rather than the participant’s official administrative documentation of immigration status. This was determined by the answer to the question: “Were you born in the United States?”. According to NHANES III designation, participants living in central counties of metropolitan areas of 1 million people or more, or fringe counties of metropolitan areas of 1 million people or more, were classified as urban. All others were classified as rural. Sampling was further divided into four country regions. Region 1 defined the Northeast, region 2, the Midwest, region 3, the South, and region 4, the West. Dog and cat ownership was determined by self report. Toxocariasis prevalence was uniform across all levels of age by any categorization of age and so was not included in the model described below to preserve parsimony. A total of 16,226 adult participants at least 17 years of age at the time of examination underwent *Toxocara* serology assessment. Though children aged 6 to 16 were also available in NAHENS III, we did not include this group as doing so would preclude the use of education level as the indicator of socioeconomic status in this study. The 15,682 sero-surveyed participants with complete data on all sociodemographic factors comprised the analytic sample for this investigation.

Census tract data were obtained from two sources. The shapefile for the census tract polygons was obtained from the US Census Bureau Tiger data source [12]. The year 2010 total male and female populations and the total African-American, Latino, White, and all other ethnicities for each census tract in New York City were also obtained directly from US census data using the same Tiger source [12]. The total number of immigrant and US-born individuals, as well as the proportion of individuals with less than a high school education, high school graduated, and college graduated were obtained from the American Community Survey (ACS), which is also conducted by the US Census Bureau and provides socioeconomic data at the census tract level. The FactFinder data extraction utility was used to retrieve the ACS data [13].

### Statistical Analysis

Multiple logistic regression was used to model the individual level NHANES III data for two purposes. First, it provides adjusted odds ratios (OR) for each risk factor included in the model and therefore provides the measure of association between each risk factor and toxocariasis adjusted for all other risk factors. Second, the predicted probabilities of toxocariasis can then be obtained for each factor level (i.e. subpopulation) included in the model. Based on this model, we computed the probability of infection for each gender, ethnicity, education, and nativity level. The form of our logistic regression model is as follows:
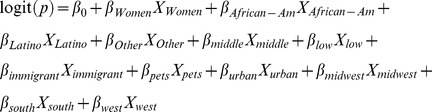
where the logit represents the log odds of toxocariasis as a function of gender, ethnicity, education level, nativity, pet ownership, metropolitan residence, and regional residence. Because the predicted probabilities obtained from this model were used to estimate census tract-specific toxocariasis in New York City, all subpopulation probabilities were computed for residents of urban areas in the Northeast region as defined above in the NHANES III survey description.

The predicted probability of toxocariasis for New York City census tracts was subsequently estimated by applying the predicted probabilities from the above multiple logistic regression model to the observed sociodemographic tract proportions obtained from the US census and the American Community Survey. The infection probability for each tract was estimated by taking a weighted average of the predicted probabilities for each subpopulation in the model. The weighted probability of toxocariasis for each subpopulation was, accordingly, weighted by the proportion of that subpopulation's presence in a given tract. First, the predicted probabilities for each subpopulation were calculated:
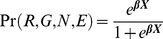
where the Pr(*R*,*G*,*N*,*E*) represent the probabilities for each subpopulation obtained from the multiple logistic regression model of the NHANES III data. For example, this model identifies a 14.5% probability of toxocariasis for US-born African-American males with a college degree. Similarly, the probability for each subpopulation has been computed for all levels of ethnicity (R), gender (G), nativity (N), and education (E). Moreover, these probabilities are estimates for urban residents in the Northeast region only. Having estimated these subpopulation-specific probabilities, the estimates were first weighted by the proportion of each education level *E* in each census tract (*w_E_*):



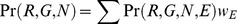
where the Pr(*R*,*G*,*N*) represent the probabilities of toxocariasis for each subpopulation of ethnicity (R), gender (G), and nativity (N), after having weighted Pr(*R*,*G*,*N*,*E*) by the proportion of each level of education (low, middle, and high) and then summing over these weighted estimates. Subsequently, Pr(*R*,*G*,*N*) were weighted by the nativity proportions *N* in each census tract (*w_N_*):



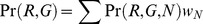
where the Pr(*R*,*G*) represent the probability of toxocariasis for each subpopulation of ethnicity (R) and gender (G), after having weighted Pr(*R*,*G*,*N*) by the proportion of immigrants and US-born in each census tract and then summing over these weighted estimates. We follow the same weighting process for gender:



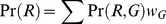
and ethnicity:



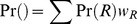
which yields the overall probability estimate, Pr(), for each census tract based on the toxocariasis probability estimates for each subpopulation from the multiple logistic regression model and the proportion of each of these subpopulations present in 2010 according to the US census. Pet ownership did not define additional subpopulations for the probability estimates as this was not available from the census data. Moreover, it was not significantly independently associated with toxocariasis. As such, pet ownership is simply controlled for in the multiple logistic regression model.

A choropleth map was then created to identify the spatial distribution of the overall predicted probability of toxocariasis, Pr(), across all census tracts of New York City. Quartiles of toxocariasis probability were used as the cutoffs for the choropleth categories. Census tracts with less than 50 residents comprised city parks and cemeteries and were considered to have zero probability of toxocariasis. For comparison with the toxocariasis map, similar choropleth maps were created to present the spatial distribution of ethnicity, nativity, and education level across all census tracts. A global Moran's index was computed to determine whether significant spatial clustering of toxocariasis probability exists across New York City. Additionally, the local Moran's index was computed to identify significant hotspots of infection probability. Stata v.11 (Stata Corp) was used to fit the multiple logistic regression model and estimate the predicted probabilities of toxocariasis for each subpopulation using the *svy* form for the *logit* command to accommodate the complex survey design employed by NHANES III. R 3.0.2 was used to compute the weighted averages for all subpopulations, estimate the census tract toxocariasis probabilities, map these estimates using the choropleth() function in the GISTools package, and estimate the global Moran's index using the spdep package (www.r-project.org). The map of the local Moran’s index was created in ArcGIS v. 10 (ESRI).

## Results

The form of the fitted regression model is represented in Table 1. Odds ratios (OR) are presented for each risk factor in the model. Gender, ethnicity, education level, nativity, urban residency, and region residency are all associated with the seroprevalence of *Toxocara* spp. infection in NHANES III. In particular, African-Americans showed a 64% greater odds of toxocariasis (OR = 1.64, 95% C.I. 1.35–1.99), and those classified as “other” ethnicity demonstrated over two and a half times greater odds (OR = 2.73, 95% C.I. 1.61–4.64), compared to Whites. No significant difference in odds of *Toxocara* infection was apparent between Latinos and Whites. Education level demonstrated a strong association with toxocariasis. Those with only a high school education had a 54% greater odds of infection compared to those with a university degree (OR = 1.54, 95% C.I. 1.15–2.05), while those with less than high school had almost two and a half times the odds of infection (OR = 2.49, 95% C.I. 1.86–3.32). Immigrants had a 92% greater odds of infection compared to US-born individuals (OR = 1.92, 95% C.I. 1.50–2.47), suggesting a strong association also exists between toxocariasis and place of nativity. As expected, women had a 28% lesser of odds of infection compared to men (OR = 0.72, 95% C.I. 0.62–0.84). There were important geographic distinctions with respect to toxocariasis prevalence as well. First, the Midwest and West regions demonstrated lesser toxocariasis prevalence compared to the Northeast, while the South exhibited no significant prevalence difference with the Northeast. Accordingly, the Northeast and South regions of the country stood out as the areas of greatest toxocariasis prevalence, which corroborates a previous report [8]. Second, urban residence was associated with lesser odds of infection compared to rural residence (OR = 0.79, 95% C.I. 0.66–0.95). Interestingly, dog or cat ownership was not associated with toxocariasis in the NHANES III sample.

**Table 1:** Odds ratios measuring the association between toxocariasis and each risk factor adjusted for all other risk factors as derived from a multiple logistic regression model of the NHANES III survey data.

Risk Factor	Odds ratio	*p*-value	95% Confidence Interval
Female (vs. Male)	0.72	<0.001	0.62–0.84
African-American (vs. White)	1.64	<0.001	1.35–1.99
Latino (vs. White)	0.86	0.304	0.64–1.15
Other (vs. White)	2.73	<0.001	1.61–4.64
Immigrant (vs. US-born)	1.92	<0.001	1.50–2.47
High School graduate (vs. College graduate)	1.54	0.004	1.15–2.05
Less than High School (vs. College graduate)	2.49	<0.001	1.86–3.32
Urban residence (vs. rural residence)	0.79	0.013	0.66–0.95
Region 2 (vs. Northeast US)	0.70	0.017	0.53–0.94
Region 3 (vs. Northeast US)	0.98	0.875	0.75–1.28
Region 4 (vs. Northeast US)	0.52	0.001	0.36–0.77
Dog or cat present (vs. absent)	1.04	0.547	0.90–1.21

**Figure 1:**
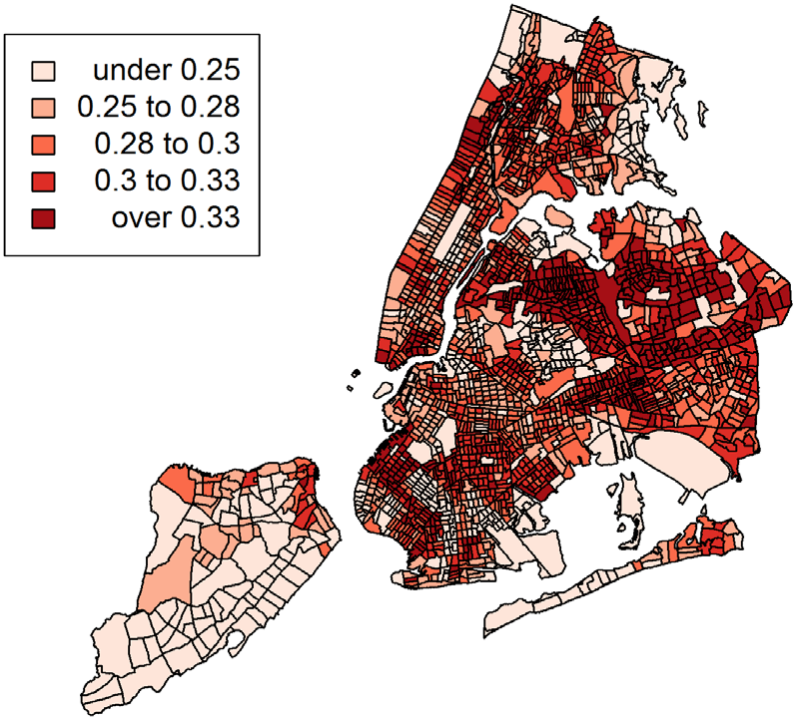
Predicted probabilities of toxocariasis by New York City census tracts. Quartiles of toxocariasis probability were used as the cutpoints for the choropleth categories. The infection probability for each tract was estimated by taking a weighted average of the predicted probabilities for each subpopulation in the logistic regression model, weighted by the proportion of that subpopulation's presence in a given census tract.

**Figure 2:**
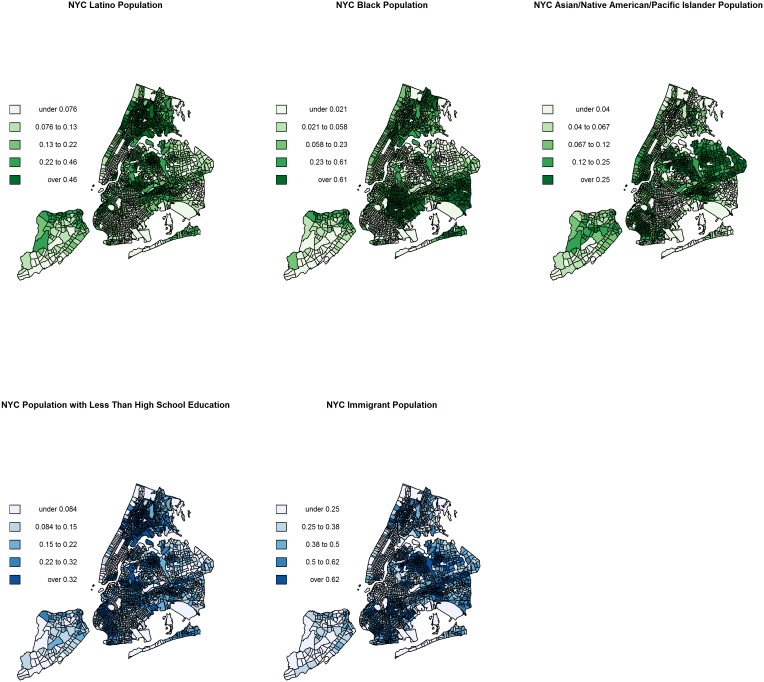
Proportions of Latino, African-American, and Asian/Native American/Pacific Islander, less than high school education, and immigrant populations in each census tract. The sociodemographic data used to compute these proportions were obtained from the US Census Bureau.

**Figure 3:**
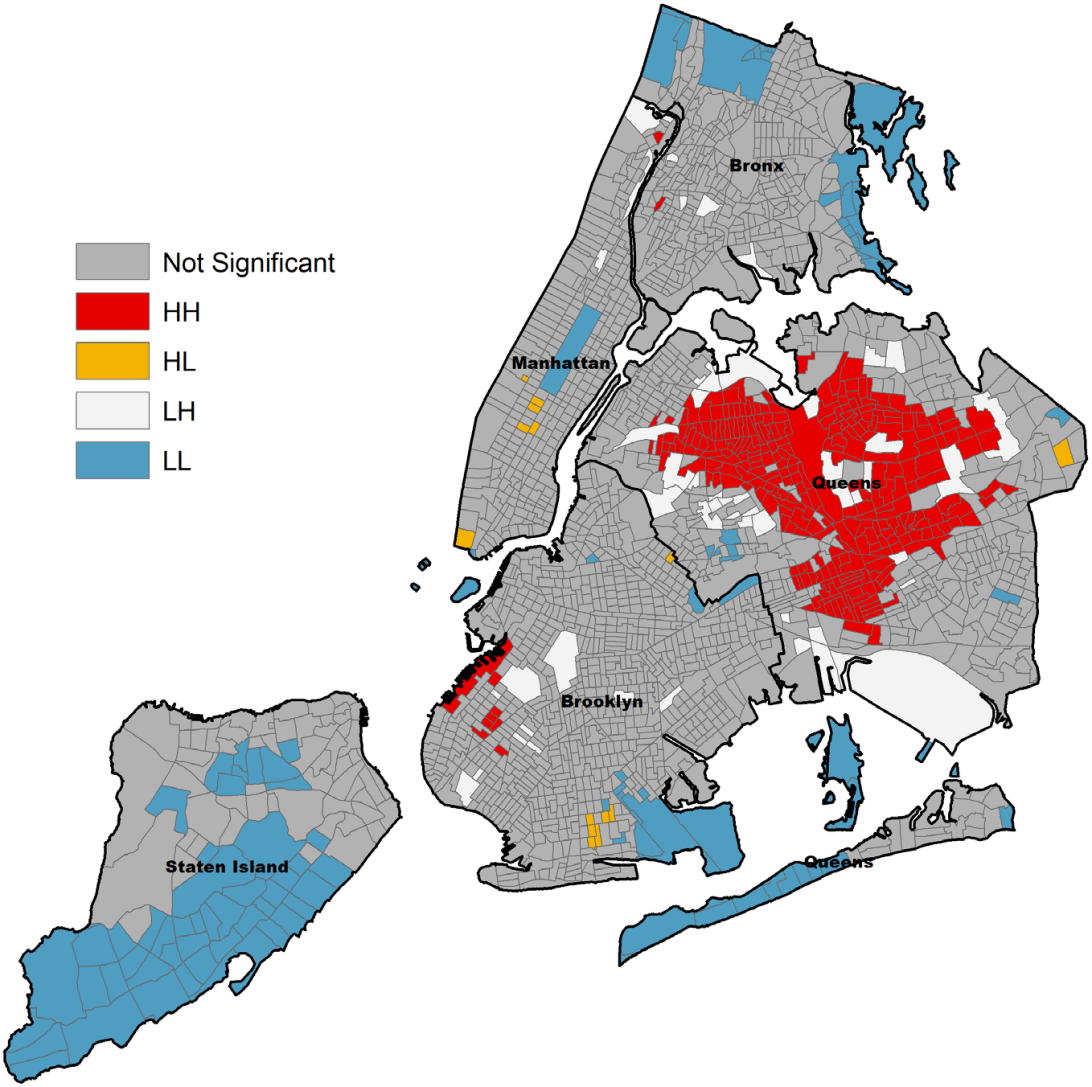
Local Moran’s index for each census tract. Hotspots identifying significantly high predicted probability of toxocariasis are highlighted in red (HH). Areas of significantly low probability of toxocariasis are highlighted in blue (LL).Census tracts with high probability surrounded by low probability tracts are highlighted in orange (HL), while tracts of low probability surrounded by high probability tracts are highlighted in white (LH). Areas of non-significant clustering are colored gray.

The associations from this multiple logistic regression model generally confirm previous reports for these risk factors based on the NHANES III data [3], [8], with minor differences due to limiting the current analysis to adults only as described in the Methods section. However, our purpose was to apply the estimates from the above model to the unique sociodemographic composition of New York City and map the city’s toxocariasis probability surface. Table 2 presents the specific predicted probability estimates from the logistic regression model for each subpopulation. The predicted probability of toxocariasis ranged from 6% among US-born Latina women with a university education to 57% among immigrant men of “other” ethnicity with less than a high school education. As expected, the same patterns of disparity suggested by the measures of association described above were apparent in these probability estimates. For example, Whites and Latinos consistently demonstrated a lower probability of toxocariasis compared to African-Americans and those classified as “other” ethnicity, even across education level, nativity, and gender. Similarly, estimates of infection probability were consistently higher for those of lower education, those born outside of the US, and men. These estimates were then weighted by the proportions of each subpopulation in each census tract and mapped in Figure 1. The map identifies those areas estimated to have a high probability of toxocariasis based on 1) the risk factors identified by the NHANES III sample, and 2) the specific sociodemographic composition of each census tract in New York City. For comparison with the spatial distribution of infection probability, Figure 2 displays a lattice of maps of the distribution of the proportion of ethnic groups, low education level, and immigrants, respectively, by census tract. The global Moran's index was 0.41 (p-value<0.00001) suggesting that there is significant spatial clustering of the predicted probability of toxocariasis across New York City. Figure 3 displays the high probability hotspots in red, with large sections in Queens and a small section in Brooklyn demonstrating the highest probability of toxocariasis based on the sociodemographic composition of these areas.

**Table 2:** Predicted probabilities of toxocariasis for each subpopulation estimated from the multiple logistic regression model of the NHANES III survey data.

Subpopulation	Estimated Probability	Linearized Standard Error
High education, US-born, White male	0.094	0.013
High education, US-born, African-American male	0.145	0.024
High education, US-born, Latino male	0.082	0.014
High education, US-born, Other male	0.220	0.051
High education, US-born, White female	0.069	0.010
High education, US-born, African-American female	0.109	0.019
High education, US-born, Latino female	0.060	0.012
High education, US-born, Other female	0.169	0.041
High education, Immigrant, White male	0.164	0.026
High education, Immigrant, African-American male	0.244	0.039
High education, Immigrant, Latino male	0.145	0.022
High education, Immigrant, Other male	0.350	0.070
High education, Immigrant, White female	0.125	0.021
High education, Immigrant, African-American female	0.190	0.032
High education, Immigrant, Latino female	0.109	0.018
High education, Immigrant, Other female	0.280	0.061
Middle education, US-born, White male	0.137	0.013
Middle education, US-born, African-American male	0.206	0.023
Middle education, US-born, Latino male	0.120	0.018
Middle education, US-born, Other male	0.302	0.059
Middle education, US-born, White female	0.103	0.008
Middle education, US-born, African-American female	0.158	0.017
Middle education, US-born, Latino female	0.090	0.014
Middle education, US-born, Other female	0.238	0.049
Middle education, Immigrant, White male	0.232	0.024
Middle education, Immigrant, African-American male	0.332	0.033
Middle education, Immigrant, Latino male	0.207	0.022
Middle education, Immigrant, Other male	0.453	0.070
Middle education, Immigrant, White female	0.180	0.017
Middle education, Immigrant, African-American female	0.265	0.027
Middle education, Immigrant, Latino female	0.159	0.018
Middle education, Immigrant, Other female	0.374	0.063
Low education, US-born, White male	0.204	0.017
Low education, US-born, African-American male	0.296	0.028
Low education, US-born, Latino male	0.180	0.025
Low education, US-born, Other male	0.411	0.066
Low education, US-born, White female	0.156	0.010
Low education, US-born, African-American female	0.232	0.020
Low education, US-born, Latino female	0.137	0.020
Low education, US-born, Other female	0.335	0.057
Low education, Immigrant, White male	0.328	0.030
Low education, Immigrant, African-American male	0.444	0.036
Low education, Immigrant, Latino male	0.295	0.028
Low education, Immigrant, Other male	0.571	0.067
Low education, Immigrant, White female	0.260	0.021
Low education, Immigrant, African-American female	0.366	0.030
Low education, Immigrant, Latino female	0.233	0.023
Low education, Immigrant, Other female	0.490	0.065

Because these predicted probabilities were subsequently applied to New York City census tracts, all subpopulation probabilities were computed based on residents of urban areas in the Northeast region.

## Discussion

Applying the only source of population-based, region- and urban-specific toxocariasis prevalence estimates in the US to census tract population data in New York City, the current study derived the first predicated probability surface of toxocariasis for a major US city. The map identifies areas of high infection probability and their spatial variation based on the sociodemographic composition of this city. This probability surface does not provide a definitive profile for this important neglected infection of poverty in New York City. Rather, the utility of this map is to provide direction for targeted sampling of the city to identify areas of high risk based on locally acquired samples. It is intended to guide the study of risk rather than define risk itself.

Comparing the risk surface map to the maps of ethnicity, nativity and immigration, there are some interesting features that may distinguish the relevance of different demographic factors to the potential spatial distribution of toxocariasis in New York City. For example, while African-Americans, in general, had a relatively high predicted probability of toxocariasis, areas with high proportions of African-Americans were not estimated to be universally high in the toxocariasis map, although these areas did exhibit higher infection probability compared to areas with high proportions of Whites. In addition, although the probability of toxocariasis was low for Latinos overall, some areas with high proportions of Latinos, such as the south Bronx and northern Manhattan, were associated with relatively high infection probability in the map. If we also consider the maps of nativity and education in comparison with the toxocariasis probability surface, we can see that the spatial patterns of the subpopulations defined by nativity and education levels influence the patterns of toxocariasis probability. Clearly, a single demographic such as ethnicity is insufficient to predict the probability of toxocariasis in a place as demographically complex as New York City.

To date, toxocariasis surveillance of the general population has not been undertaken in New York City. Moreover, following the nationwide NHANES III surveillance completed in 1994 there have been no toxocariasis surveillance programs developed in any local regions in the US and only a handful of clinic-based studies conducted. One serologic study conducted in New York City between 1980 and 1981 identified an overall toxocariasis prevalence of 5.4% [6]. However, this sample was comprised primarily of young children (72% were ≤5 years of age) who were part of a lead screening program. This seroprevalence estimate, therefore, cannot be extrapolated to the general New York City population. In the current investigation, we followed a similar approach to that of Congdon and Lloyd [8]. Their investigation applied NHANES III data to county sociodemographic data to estimate the probability of toxocariasis across the US at the county level. That study provided a good estimate of the spatial heterogeneity of toxocariasis across the US as a whole and identified regions of high infection probability. However, these estimates are too small in scale to describe the toxocariasis probability surface across a single city. Moreover, while appropriate for their purposes, that report did not account for place of nativity, which is essential for any probability estimates in New York City where the immigrant population comprises 37% of the total.

The multiple logistic regression model used to compute the predicted probabilities of toxocariasis was based on NHANES III data that have inherent limitations warranting further discussion. First, while all the subpopulations identified in the NHANES III sample were equally represented in the census data for New York City, NHANES III classified ethnicity more crudely than did the US Census Bureau. African-Americans, Mexican-Americans, Latinos, and Whites were all clearly identified in the NHANES III survey, while anyone not of these four ethnic categories was classified as “other”. The other category comprised very substantive groups of people, most prominently, Asians. Therefore, applying the subpopulation predicted probabilities from the multiple logistic regression model to the subpopulation proportions in each census tract was ethnically precise for African-Americans, Mexican-Americans, Latinos, and Whites but necessarily vague for those of other ethnicities, which, again, would be expected to be predominantly Asians even though this category also specifically included Native Americans and Pacific Islanders. This is further reflected in Figure 2 as many of those sections identified with high proportions of “other” are areas with high proportions of Asians. Second, NHANES III was completed in 1994. Toxocariasis prevalence may have indeed changed during the 16 year period between 1994 and 2010. However, the rigorous study design employed by NHANES III ensured a representative sample of the US population in distinct well-defined geographic locations and, as such, the measures of association derived from this study are themselves good estimates for the risk factors and subpopulations included. Moreover, the use of education level as an indicator of socioeconomic status in the current study is likely to be more robust across time than other purely economic measures such as measures of income. Nevertheless, it is possible that changes in the socioeconomic status of different ethnic groups over time may subsequently alter toxocariasis risk in those groups, thus potentially biasing the predicted probabilities. Here again, we feel any such bias would be minimal because the distributions of ethnicity and education level in New York City in 2010, as presented in this study, were highly correlated and reflect similar patterns of disparity present at the time of NHANES III. Therefore, given the complete lack of any city-wide population-based estimates for New York City, the investigators feel there is an urgent need for small-area estimation of toxocariasis based on the proven rigorous survey methodology demonstrated by NHANES III. The probability surface produced is thus intended to identify potential high and low probability areas so that such areas may be highlighted for future efforts at targeted serologic surveys of the population and environmental sampling for ova across New York City.

In conclusion, this study applied predicted probabilities obtained from a large, representative population-based survey of the US population, to the sociodemographic configuration of census tracts in New York City to estimate the probability of toxocariasis and the spatial patterns that emerge from the city's diverse communities. This probability map cannot define toxocariasis risk for New York City. However, using the best surveillance data we currently have in the US, we can identify subpopulations with a high occurrence of infection and then identify similar populations within New York City to estimate the distribution of the probability of toxocariasis in this local setting. Because this study estimates infection probability at this larger, local scale it has more utility in directing locally targeted surveillance efforts than would country-wide estimates. The map provides locations of potentially high and low toxocariasis occurrence, which can then be surveyed more efficiently, rather than unguided random sampling that would be considerably more expensive and may be an inefficient use of resources. These results should be used to inform the future development and implementation of much needed surveillance of a significant neglected infection of poverty in the United States.
